# Cancer-Associated Fibroblasts Build and Secure the Tumor Microenvironment

**DOI:** 10.3389/fcell.2019.00060

**Published:** 2019-04-24

**Authors:** Tianyi Liu, Linli Zhou, Danni Li, Thomas Andl, Yuhang Zhang

**Affiliations:** ^1^Division of Pharmaceutical Sciences, College of Pharmacy, University of Cincinnati, Cincinnati, OH, United States; ^2^College of Chemistry and Chemical Engineering, Guangxi University for Nationalities, Nanning, China; ^3^Burnett School of Biomedical Sciences, University of Central Florida, Orlando, FL, United States

**Keywords:** cancer-associated fibroblast, tumor microenvironment, extracellular matrix, therapy, mechanoreciprocity

## Abstract

Tumor cells reside in a highly complex and heterogeneous tumor microenvironment (TME), which is composed of a myriad of genetically stable non-cancer cells, including fibroblasts, immune cells, endothelial cells, and epithelial cells, and a tumor-specific extracellular matrix (ECM). Cancer-associated fibroblasts (CAFs), as an abundant and active stromal cell population in the TME, function as the signaling center and remodeling machine to aid the creation of a desmoplastic tumor niche. Although there is no denial that the TME and CAFs may have anti-tumor effects as well, a great deal of findings reported in recent years have convincingly revealed the tumor-promoting effects of CAFs and CAF-derived ECM proteins, enzymes, chemical factors and other downstream effectors. While there is growing enthusiasm for the development of CAF-targeting therapies, a better understanding of the complexities of CAF-ECM and CAF-cancer cell interactions is necessary before novel therapeutic strategies targeting the malignant tumor “soil” can be successfully implemented in the clinic.

## Introduction

In the last decades, despite considerable advances in the development of novel immunotherapies and targeted therapies, no significant improvements have been made in overall survival rates for patients with malignant solid tumors. One major reason for this lack of substantial improvement is the development of drug resistance in tumor cells, which usually reveals itself within a few months after patients are treated with anti-cancer drugs. An Achilles’ heel of many current therapeutic approaches is that these therapies primarily target the fast-growing tumor “seeds” but largely ignore the fertilizing tumor “soil” – the tumor microenvironment (TME) (de Groot et al., 2017). The TME influences the penetration, distribution, and metabolism of therapeutic agents, and produces molecular factors and signals, which positively or negatively regulate how tumor cells grow, migrate and respond to therapeutic agents. As cancer-associated fibroblasts (CAFs) appear to be a major TME component in many tumors and are critical for shaping the “soil” within which the tumor cells thrive (LeBleu and Kalluri, 2018), they have become the prime target for the efforts to modify non-tumor cell behavior to suppress tumor growth. It is clear that the TME and CAFs are not always pro-tumorigenic due to the complexities of their interactions with tumor cells. However, in this review, we will mainly explore the tumor-promoting interactions between cancer cells and fibroblasts and how CAFs may be persuaded using novel therapeutic approaches to renounce their fealty to the tumor cells and even produce a tumor-suppressive “soil.”

## Stromal Fibroblasts, Myofibroblasts, and CAFs

Tumors are often referred to as “wounds that never heal” (Dvorak, 1986) because the stroma of a wound and a tumor share many similarities, such as fibroblast activation, increased extracellular matrix (ECM) protein production and intensive remodeling processes (Foster et al., 2018). Activated stroma is molecularly, biochemically and pathologically different from the normal stroma. In the stroma of normal human skin, fibrous proteins fill in the interstitial space between stromal fibroblasts while epithelial keratinocytes rest on the sheet-like basement membrane. Under normal physiological conditions, non-contractile fibroblasts are generally flat, spindle-shaped and recognized as quiescent and inert cells in the ECM (Valkenburg et al., 2018).

Myofibroblasts were first identified in the tissue wound repair process, during which fibroblasts or smooth muscle cells differentiate and gain a contractile phenotype (McAnulty, 2007). The major roles of myofibroblasts in wound healing are to contract the wounds and produce and organize the ECM (Darby et al., 2014). As the wound closes and heals, myofibroblasts become apoptotic and finally disappear as the scar is formed (Desmouliere et al., 1995). Myofibroblasts are different from normal fibroblasts in many aspects, including (1) ruﬄed membranes and a highly active endoplasmic reticulum (Baum and Duffy, 2011); (2) expression of alpha smooth muscle actin (α-SMA or ACTA2) and increased levels of vimentin (VIM) (Ronnov-Jessen and Petersen, 1993) and (3) formation of complex and organized stress fibers and fibronexus adhesion complexes (Rao et al., 2016). The bundles of microfilaments in myofibroblasts interact with the ECM proteins through fibronexus adhesion complexes, thereby allowing myofibroblasts to sense the tension in their surrounding microenvironment and maintain the cellular contractile force through the network of cytoskeletal proteins. As a feedback response, myofibroblasts increase matrix fibroplasia by producing ECM proteins, including collagen, elastin (ELN), fibronectin (FN1), tenascin (TNC), and remodeling enzymes, such as matrix metalloproteinases (MMPs).

Tumor growth recapitulates the basic wound healing program and shares many similarities, such as deposition and crosslinking of fibrin and FN1 and the recruitment of immune cells (Schafer and Werner, 2008). However, unlike a normal healing wound, which is restricted to a certain area and proceeds directionally through the steps of hemostasis, inflammation, proliferation, and maturation/remodeling, cancer cells distort the wound healing program and have the potential to migrate away or expand from the initiation site and invade adjacent tissues. CAFs are the fibroblasts found in the stroma of human cancers but differ from normal fibroblasts in their increased collagen and ECM protein production and up-regulated secretion of pro-tumor factors (Bauer et al., 2010; Xing et al., 2010; Pidsley et al., 2018). There are several important sources from which CAFs could be derived, including: (i) recruitment and activation of resident fibroblasts (Fukino et al., 2004); (ii) epithelial-mesenchymal transition (EMT) of resident epithelial cells (Petersen et al., 2001); (iii) endothelial to mesenchymal transition (EndMT) of resident endothelial cells (Zeisberg et al., 2007a,b) and (iv) differentiation of bone marrow mesenchymal cells (Quante et al., 2011). In a sense, CAFs or at least a subset of CAFs are wound-like myofibroblasts that mediate a deranged chronic wound healing program in tumors. For example, a large part of CAFs share similar features as α-SMA-positive (α-SMA+) myofibroblasts (Shiga et al., 2015). In addition, other than myofibroblastic CAFs, subpopulations of CAFs without α-SMA expression have also been reported, e.g., in pancreatic cancer (Ohlund et al., 2017).

## Heterogeneity of Stromal Fibroblasts, Myofibroblasts, and CAFs

Understanding the state, complexity and heterogeneity of normal fibroblasts may shed light on the origins of CAFs, how they form and how their transdifferentiation may be regulated in the early stages of tumorigenesis and at the tumor front. Two major populations of fibroblasts in the human dermis are papillary and reticular fibroblasts, which possess distinct morphology, molecular expression, and cellular functions (Harper and Grove, 1979). Janson et al. (2012) performed gene expression analysis on cultured papillary and reticular fibroblasts and identified 116 differentially expressed genes. However, except for matrix Gla protein (MGP), which is almost exclusively expressed in the reticular dermis, they did not discover any *in vivo* markers to separate the two fibroblast populations. Korosec et al. (2019) performed lineage identity and location studies of human dermis using two markers, fibroblast activation protein (FAP) and THY1 (Cluster of Differentiation 90 or CD90). They found that papillary fibroblasts are FAP^+^; THY1^-^, whereas FAP^-^; THY1^+^ fibroblasts are mainly of the reticular lineage. Their data showed papillary and reticular fibroblasts are not completely separated according to their spatial location.

However, recent studies have suggested that there exist more functionally distinct fibroblast subpopulations within the human dermis. A single-cell RNA sequencing (scRNA-seq) study by Philippeos et al. (2018) showed that there are five distinct fibroblast populations in adult human skin, which can be separated based on the expression of cell surface markers, including THY1, CD39, CD26 (DPP4), and regulator of G protein signaling 5 (RGS5), and are not spatially segregated. Tabib et al. (2018) performed single-cell transcriptomal analysis of cells obtained from whole skin without pre-purifying fibroblast populations. They identified two major fibroblast populations based on the expression of SFRP2/DPP4 and FMO1/LSP1 markers and five minor cell populations using CRABP1, COL11A1, PRG4, ANGPTL7, and SFRP4. In addition, there are several subpopulations in each major fibroblast population. These scRNA-seq data showed a complex and heterogeneous picture of fibroblast composition and functionality in the human dermis, which is simply beyond our original understanding of skin fibroblasts. Nevertheless, it remains to be understood how these subpopulations of fibroblasts react to either wounding or the tumorigenic process and evolve into myofibroblasts or CAFs.

Local fibroblasts are the most common origin of myofibroblasts (Hinz et al., 2007). However, several other cell types are able to differentiate into myofibroblasts, including smooth muscle cells or pericytes (Hinz et al., 2007). Fibrocytes, for example, can differentiate into myofibroblasts in skin, liver and lung tissues (Mori et al., 2005; Iwaisako et al., 2012; Ashley et al., 2017). In the liver, hepatic stellate cells are the source of myofibroblasts in liver fibrosis (Wells and Schwabe, 2015). Because of the nature of its diverse origins, myofibroblasts appear to be a heterogeneous group as well. α-SMA is the most commonly used marker to identify myofibroblasts (McAnulty, 2007). In addition, extra domain A fibronectin (EDA-FN), periostin (POSTN) and prolyl-4-hydroxylase (P4HB) have also been suggested as potential markers for myofibroblasts (Moore-Morris et al., 2014; Ngo et al., 2014; Kanisicak et al., 2016). A recent study proposed that amine oxidase, copper containing 3 (AOC3) and homeobox protein NKX2-3 are two biomarkers of pericryptal myofibroblasts in the colon and rectum (Hsia et al., 2016). Furthermore, markers that stain stromal fibroblasts can also be used to stain myofibroblasts, such as platelet derived growth factor receptor alpha (PDGFRA), THY1, and VIM, although they are not specific for myofibroblasts (Matthijs Blankesteijn, 2015).

Just like normal fibroblasts, CAFs appear to be a heterogeneous group of cells with different origins and different functions. This similarity was manifested by a study reported by Lambrechts et al. (2018). By performing scRNA-seq of 52,698 stromal cells isolated from human lung tumors and comparing with matching non-malignant lung samples, the authors identified five distinct types of fibroblasts in lung tumors, which all express their own unique set of collagens and ECM proteins that are different from non-malignant fibroblasts. Using a three-dimensional (3D) co-culture platform, Ohlund et al. (2017) identified two distinct populations of myofibroblasts and inflammatory fibroblasts in pancreatic ductal adenocarcinoma (PDA). More recently, the obscurity in CAF characterization has been further addressed by efforts to determine the exact composition of human tumor tissues using scRNA-seq. scRNA-seq data derived from head and neck squamous cell carcinoma (HNSCC) suggested that tumor myofibroblasts and CAFs may represent distinct fibroblast subpopulations (Puram et al., 2017). Overall, the authors were able to detect, in addition to normal fibroblasts and myofibroblast-like cells, two subsets of CAFs depending on the expression of FAP, THY1, connective tissue growth factor (CTGF) and podoplanin (PDPN). In their study, tumor myofibroblasts were identified based on the expression of α-SMA, melanoma cell adhesion molecule (MCAM), myosin light chain kinase (MYLK), and myosin light chain 9 (MYL9). Interestingly, scRNA-seq of colorectal cancer samples also revealed at least three fibroblast populations (Li et al., 2017). One population can be described as normal fibroblasts, the second one as myofibroblasts, which are positive for α-SMA, transgelin (TAGLN) and PDGFA, and the third one as a CAF population that is characterized by MMP2, decorin (DCN) and collagen type I alpha 2 (COL1A2). The authors determined that a key signaling pathway emanating from CAFs/myofibroblasts is transforming growth factor beta (TGF-β)/INHBA signaling, ascertaining that CAFs are not just ECM-producing factories. The scRNA-seq results of fibroblast populations are in good accordance with attempts to characterize CAFs using fluorescence activated cell sorting (FACS) (Costa et al., 2018). Such efforts in human breast cancer using six CAF markers, including FAP, integrin beta 1 (ITGB1), α-SMA, FSP1, platelet derived growth factor receptor beta (PDGFRB), and caveolin-1 (CAV1), allowed the authors to identify four distinct CAF populations, of which some were preferentially present in subsets of breast cancers. Two of the CAF populations expressed α-SMA and probably represent myofibroblast-like cells. However, a comparison of the two α-SMA+ populations revealed that one was similar to pericytes and expressed MCAM and a gene signature related to the regulation of actin cytoskeleton and muscle contraction. The second α-SMA+ population exhibited an immune-regulatory gene signature. These CAFs can function as immune-suppressors and regulators of T lymphocytes and create an immunosuppressive environment through a multi-step mechanism (Costa et al., 2018).

scRNA-seq studies of CAFs have suggested that CAF subtypes could be attributed to their origin in spatial subgroups of normal fibroblasts (Philippeos et al., 2018; Tabib et al., 2018). However, Biffi et al. (2019) reported that tumor-secreted TGF-β/ and IL1 can promote CAF heterogeneity. Subsets of CAFs can function to either support or suppress tumor cells. For example, it was reported that cancer cells undergo the EMT process and acquire invasive phenotypes through the activation of the TGF-β-SMAD signaling pathway induced by CAFs (Bellomo et al., 2016). In addition, by producing pro-angiogenic factors, such as fibroblast growth factor 2 (FGF2) and VEGFA (De Palma et al., 2017), CAFs regulate angiogenesis in the stroma, thereby providing essential nutrients for highly proliferative tumor cells. CAFs can also assist tumor cells in overcoming immune surveillance by recruiting immunosuppressive cells, such as M2 macrophages and myeloid-derived suppressor cells (MDSC) (Flavell et al., 2010; Yang et al., 2016). However, it was reported that ablation of subsets of α-SMA+ CAFs in PDA could result in a more aggressive cancer phenotype and reduced animal survival (Ozdemir et al., 2014). In summary, the heterogeneity of CAFs reflects the diversity and complexity of the TME, and more careful research is needed to fully comprehend the interactions among CAFs, tumor cells and the ECM.

## CAF-Derived ECM Proteins

**FIGURE 1:**
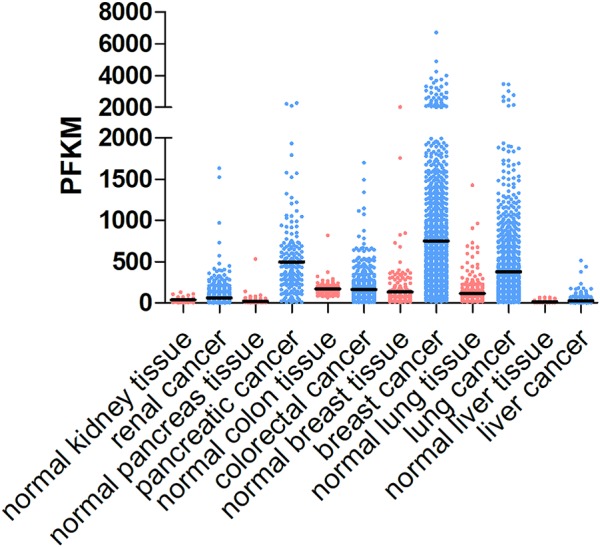
COL1A1 expression levels in different cancer types and corresponding normal tissues. COL1A1 expression levels vary in different types of cancer, including renal cancer, pancreatic cancer, colorectal cancer, breast cancer, lung cancer, and liver cancer and generally are higher in tumor tissues than those in normal tissue. The data are obtained from https://www.proteinatlas.org/ENSG00000108821-COL1A1/tissue and https://www.proteinatlas.org/ENSG00000108821-COL1A1/pathology.

The tumor ECM is composed of a complex mixture of macromolecules, including fibrous proteins (collagen, ELN), proteoglycans (heparan sulfate, chondroitin sulfate), glycosaminoglycans (hyaluronic acid), and glycoproteins (FN1, laminins, TNC) (Botti et al., 2013). ECM proteins are not just bystanders of the tumorigenic process. Instead, they provide structural signals and support for tumor cells to grow and migrate. Although many other stromal cell types and tumor cells can also produce ECM proteins, CAFs appear to be the major player in the stroma that synthesizes, secrets, assembles and modifies the ECM composition and organization (Faouzi et al., 1999; Yoshimura et al., 2015; Erdogan et al., 2017). For example, elevated collagen production and crosslinking have been coupled with increased tumor stiffness and progression. It was estimated that fetal rat fibroblasts synthesize about 40 molecules of procollagen/cell per second (McAnulty et al., 1991). Many cancers are characterized by elevated levels of collagen production, e.g., COL1A1 (Figure 1). Faouzi et al. (1999) reported that myofibroblasts are the primary source of collagen (types I, IV, V and VI) in the stroma of human hepatocellular carcinoma. In addition, CAF-derived laminin was shown to induce cervical cancer cell migration via the interaction with integrin α6β4 (Fullar et al., 2015). In an *in vitro* ovarian cancer spheroid model, CAF-secreted versican promoted cancer invasion in a TGF-β-dependent manner (Yeung et al., 2013).

FN1 was first found to be overexpressed in human solid tumor specimens in 1981 (Stenman and Vaheri, 1981). Although tumor cells produce FN1 themselves, stromal cells, such as CAFs, are indispensable for bulk FN1 assembly (Attieh et al., 2017; Erdogan et al., 2017). Like collagen, the pro-tumorigenic role of FN1 is also well-acknowledged. In 1998, Menzin et al. (1998) proposed that FN1 may play an important role in regulating the invasive phenotype and poor patient prognosis in ovarian cancer. FN1 was also documented to promote cell cohesion, basement membrane invasion and tumor growth in glioblastoma (GBM). Depletion of FN1 in GBM cells resulted in weaker cell-cell contact and less collective migration in an *in vitro* spheroid model, highlighting the role of FN1 as a “biological glue” (Serres et al., 2014). The role of FN1 in cell cohesion has also been observed in fibroblast spheroids. FN1-depleted fibroblasts failed to form compact spheroids *in vitro*. Furthermore, the blockade of FN1-integrin interactions impeded fibroblast activation (Salmenpera et al., 2008).

Tenascin is another highly expressed ECM glycoprotein in the tumor stroma, such as the stroma of canine mammary carcinoma, pancreatic cancer and prostate cancer, and is associated with poor patient prognosis (Yoshimura et al., 2015; Cai et al., 2017; Ni et al., 2017). Mouse embryonic fibroblasts lacking TNC have robust overexpression of tissue plasminogen activator (tPA) and increased capacity to digest fibrin *in situ* (Brellier et al., 2011). Furthermore, they discovered that there was a correlation between *in vivo* TNC expression and fibrin accumulation in head and neck squamous cell carcinomas (SCC) and lung carcinomas, further confirming that TNC functions as a regulator of the fibrinolytic system.

## CAF-Derived ECM Enzymes

Tumor progression and metastasis require a distinct ECM biomechanical architecture, for which CAFs not only produce and secrete ECM proteins and also actively participate in the ECM proteolysis, crosslinking and assembly processes. In such a rigid and highly crosslinked tumor stroma, drug penetration is one potential reason for tumor cells to escape therapy. In addition, CAF-mediated ECM remodeling is a highly responsive process of receiving, processing and responding to the cellular, molecular and mechanical signals in the TME. The lysyl oxidase (LOX) family and MMPs represent two major types of remodeling enzymes produced by CAFs. As a highly adaptive and mechanically responsive stromal cell type, CAFs sense and respond to the ECM stiffness in a LOX/MMP-dependent manner and further fine-tune the CAF-ECM interactions.

The LOX family oxidases include five members: LOX and lysyl oxidase like (LOXL) 1, 2, 3, and 4 (Wang et al., 2016). They share similar structures and catalyze the cross-linking of collagen and ELN by oxidation, contributing to increased stiffness of the tumor stroma. In tissue fibrosis, it was demonstrated that fibroblast-derived LOX could be induced by different soluble factors, such as insulin-like growth factor-binding protein 5 (IGFBP5) (Nguyen et al., 2018) and POSTN (Kumar et al., 2018), and by the transcription factor hypoxia inducible factor 2 alpha (HIF2A) (Hikage et al., 2019). Elevated levels of LOX family oxidases are often observed in cancers and play a prominent role in cancer progression. Gene expression analysis of mouse mammary tumors revealed that activated fibroblasts are the major producers of LOX family oxidases (Pickup et al., 2013). When colon cancer patient-derived CAFs and normal fibroblasts were compared by proteomic analysis, LOXL2 was found to be overexpressed in CAFs and was identified as a predictive prognostic factor in stage II colon cancer patients (Torres et al., 2015). Similarly, LOXL2 expression in gastric CAFs was also demonstrated to be positively correlated with the invasive ability of gastric cancer cells (Kasashima et al., 2014).

In breast cancer, LOXL2 inhibition showed anti-tumor effects in reducing tumor size and angiogenesis. Furthermore, a combination of LOX and LOXL2 inhibitors resulted in even smaller and less metastatic tumors (Chang et al., 2017). Interestingly, in mice bearing aggressive breast cancer, anti-LOXL2 monoclonal antibody AB0023 exhibited potent inhibitory effects in activated fibroblast as suggested by an 88% reduction of α-SMA+ cells by immunohistochemistry (IHC) after AB0023 treatment (Barry-Hamilton et al., 2010). The inhibitory effect was also shown to be closely associated with the reduction in cross-linked collagenous ECM matrix. Recently, an *in vitro* study using siRNA adenovirus vector to silence LOXL2 expression in mouse lung fibroblast also showed that the proliferation of lung fibroblasts was significantly decreased via the TGF-β/Smad signaling pathway (Wen et al., 2018). All these findings highlighted the role of CAF-derived LOX family oxidases in regulating tumor migration and invasion and potential beneficial outcomes of targeting CAF-synthesized LOX family oxidases.

The ability of cancer cells to digest surrounding ECM and localize to distal sites has long been attributed to MMPs, which are zinc-containing endopeptidases. MMPs play pivotal roles in creating the paths for tumor cells to leave the primary tumor niche and wade through the stiff matrix. There are 24 MMPs in mammals (Vandenbroucke and Libert, 2014), of which MMP2 and MMP9 are found to be overexpressed in many cancer types and promote tumor progression and metastasis (Table 1). CAFs were shown to be the major producer of MMP2 in mouse lung tumors as indicated by IHC staining results showing MMP2 primarily localizes to fibroblasts (Bates et al., 2015). Using the online database proteinatlas.org, we summarized the correlation between 13 MMPs and patient prognosis status in nine human cancers in Table 2 based on the RNA-Seq data. Four MMPs (MMP10, MMP15, MMP24, MMP25) are shown to be favorable to patient prognosis as their expression levels are positively correlated with patient survival. However, the expression levels of eight MMPs (MMP1, MMP3, MMP7, MMP11, MMP12, MMP14, MMP19, and MMP28) are shown to be negatively correlated with patient survival. Interestingly, MMP9 seemed to have context-dependent roles in different cancer types. In conclusion, the roles of different MMPs in the TME need to be carefully examined based on cancer types and stages, and this should also be one major consideration when designing, dosing and scheduling MMP-targeting drugs for cancer patients (Iyer et al., 2012).

**Table 1:** Expression of MMP2 and MMP9 is correlated with cancer progression and metastasis.

MMP2	References	MMP9	References
Basal-cell carcinoma	Gozdzialska et al., 2016	Basal-cell carcinoma	Gozdzialska et al., 2016
Brain cancer	Wang M. et al., 2003; Tabouret et al., 2014	Brain cancer	Wang M. et al., 2003; Li et al., 2016
Breast cancer	Yari et al., 2014; Chen Y. et al., 2015; Ramos et al., 2016; Tabouret et al., 2016	Breast cancer	Wu et al., 2014; Yousef et al., 2014
Colorectal cancer	Groblewska et al., 2014	Gastric cancer	Wang et al., 2014; Chen S.Z. et al., 2015
Endometrial adenocarcinoma	Li et al., 2014	Liver cancer	Sun et al., 2014
Gastric cancer	Wang et al., 2014	Lung cancer	Lee et al., 2015; Zhang et al., 2015; Gong et al., 2016; Yu et al., 2016
Lingual and gingival cancers	Nishio et al., 2016	Pancreatic cancer	Jakubowska et al., 2016
Lung cancer	Zhang et al., 2015	Pituitary adenoma	Liu et al., 2016
Melanoma	Kamyab-Hesari et al., 2014	Prostate cancer	Oguic et al., 2014
Osteosarcoma	Zhang and Zhang, 2015	Squamous cell carcinoma	Stanciu et al., 2016
Ovarian cancer	Fu et al., 2015

**Table 2:** Correlations between MMP/LOX expression and the 5-year survival rates of cancer patients^∗∗^.

MMP/LOX	Prognosis^∗^	Cancer type	5-year survival			
			High expression	Low expression	Sample size	*p*-value
MMP1	Unfavorable	Renal cancer	60%	82%	877	9.90E-10
	Unfavorable	Liver cancer	36%	50%	365	0.0000042
	Unfavorable	Cervical cancer	59%	74%	291	0.00047
MMP3	Unfavorable	Pancreatic cancer	5%	34%	176	0.00041
	Unfavorable	Cervical cancer	45%	71%	291	0.00097
MMP7	Unfavorable	Liver cancer	38%	60%	365	0.00025
	Unfavorable	Lung cancer	34%	50%	994	0.00034
MMP9	Unfavorable	Renal cancer	64%	78%	877	0.000041
	Favorable	Endometrial cancer	81%	60%	541	0.00025
	Unfavorable	Liver cancer	37%	64%	365	0.00072
MMP10	Favorable	Urothelial cancer	48%	27%	406	0.00071
MMP11	Unfavorable	Renal cancer	65%	81%	877	0.00026
MMP12	Unfavorable	Liver cancer	33%	51%	365	0.00014
MMP14	Unfavorable	Renal cancer	58%	73%	873	0.00013
	Unfavorable	Ovarian cancer	20%	35%	373	0.00095
MMP15	Favorable	Renal cancer	86%	65%	877	8.50E-08
	Favorable	Urothelial cancer	49%	30%	406	0.00031
MMP19	Unfavorable	Renal cancer	62%	81%	877	8.60E-09
MMP24	Favorable	Renal cancer	74%	51%	877	8.20E-11
MMP25	Favorable	Head and neck cancer	51%	29%	499	0.000011
MMP28	Unfavorable	Pancreatic cancer	16%	40%	176	0.0000063
LOX	Unfavorable	Renal cancer	64%	87%	877	3.90E-08
	Unfavorable	Urothelial cancer	25%	51%	406	0.00033
	Unfavorable	Liver cancer	36%	52%	365	0.00074
LOXL1	Unfavorable	Glioma	0	10%	153	0.00013
LOXL2	Unfavorable	Lung cancer	31%	57%	994	1.50E-07
	Unfavorable	Renal cancer	54%	72%	877	2.90E-07
	Unfavorable	Cervical cancer	52%	82%	291	0.0000098
	Unfavorable	Glioma	0	10%	153	0.00018
	Unfavorable	Pancreatic cancer	6%	43%	176	0.00091
LOXL3	Unfavorable	Renal cancer	61%	77%	877	8E-08
LOXL4	Unfavorable	Glioma	2%	16%	153	0.00054
	Unfavorable	Ovarian cancer	23%	39%	373	0.00096

*^∗^The prognosis of each group of patients was examined by Kaplan–Meier survival estimators, and the survival outcomes of the two groups were compared by log-rank tests.*

*^∗∗^Data available from:*

*MMP1: https://www.proteinatlas.org/ENSG00000196611-MMP1/pathology*

*MMP3: https://www.proteinatlas.org/ENSG00000149968-MMP3/pathology*

*MMP7: https://www.proteinatlas.org/ENSG00000137673-MMP7/pathology*

*MMP9: https://www.proteinatlas.org/ENSG00000100985-MMP9/pathology*

*MMP10: https://www.proteinatlas.org/ENSG00000166670-MMP10/pathology*

*MMP11: https://www.proteinatlas.org/ENSG00000099953-MMP11/pathology*

*MMP12: https://www.proteinatlas.org/ENSG00000262406-MMP12/pathology*

*MMP14: https://www.proteinatlas.org/ENSG00000157227-MMP14/pathology*

*MMP15: https://www.proteinatlas.org/ENSG00000102996-MMP15/pathology*

*MMP19: https://www.proteinatlas.org/ENSG00000123342-MMP19/pathology*

*MMP24: https://www.proteinatlas.org/ENSG00000125966-MMP24/pathology*

*MMP25: https://www.proteinatlas.org/ENSG00000008516-MMP25/pathology*

*MMP28: https://www.proteinatlas.org/ENSG00000271447-MMP28/pathology*

*LOX: https://www.proteinatlas.org/ENSG00000113083-LOX/pathology*

*LOXL1: https://www.proteinatlas.org/ENSG00000129038-LOXL1/pathology*

*LOXL2: https://www.proteinatlas.org/ENSG00000134013-LOXL2/pathology*

*LOXL3: https://www.proteinatlas.org/ENSG00000115318-LOXL3/pathology*

*LOXL4: https://www.proteinatlas.org/ENSG00000138131-LOXL4/pathology*

## CAF-ECM Interactions

**FIGURE 2:**
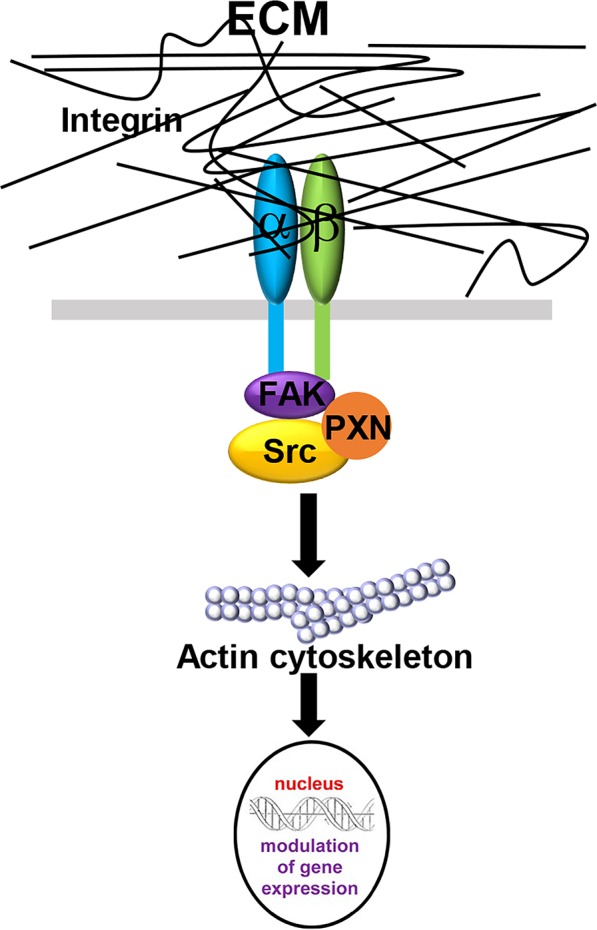
The “outside-in” signaling mode in CAF-ECM interactions. When the membrane-bound integrin receptors interact with ECM proteins, integrin α- and β- subunits dimerize to activate downstream FAK1 and Src signaling and induce cytoskeleton remodeling, thereby converting external mechanical signals into cellular and biochemical signals inside the cells.

The interactions between CAFs and the ECM influence the stiffness of the tumor stroma and can be described using the term “mechanoreciprocity” (van Helvert et al., 2018), which consists of both “outside-in” and “inside-out” signaling modes (Shattil et al., 2010). The “outside-in” signaling mode is a well-established mechanism, by which ECM proteins can function as ligands and bind to integrin receptors on the cell membrane (Table 3). Integrins are transmembrane receptors composed of a heterodimer of α and β subunits. As shown in Figure 2, when the cells encounter a rigid ECM, the integrin molecules become dimerized to trigger the focal adhesion cascade and the activation of downstream signaling, including tyrosine protein kinase SRC and focal adhesion kinase FAK1, thereby converting external mechanical signals into cellular and biochemical signals inside the cells (Barczyk et al., 2010; Tucker and Chiquet-Ehrismann, 2015; Benito-Jardon et al., 2017). Integrin α11β1 is a stromal cell-specific receptor for collagen and also known as an important regulator for fibroblast activation (Carracedo et al., 2010). Zhu et al. (2007) showed that the growth of the tumors formed by non-small-cell lung carcinoma (NSCLC) cell lines, A549, NCI-H460, and NCI-H520 mixed with integrin α11-deficient fibroblasts were significantly impeded as compared with the tumors derived from the mixture of either tumor cell line and wild-type fibroblasts. In this case, fibroblasts, originally good “listeners” and “responders” to the mechanical cues, lost their active ECM remodeling ability after the fibroblast-ECM interaction was blocked. In another example, cardiac fibroblasts cultured on a stiff matrix expressed increased amounts of LOX, further crosslinked collagen fibers and stiffened the ECM. To the contrary, the inhibition of the binding between α2β1 integrin and collagen I ablated this effect and downregulated LOX expression (Gao et al., 2016). In the “outside-in” signaling mode, the mechanical cues can also activate other signaling pathways in fibroblasts, such as the mitogen-activated protein kinase (MAPK) pathway (Wang J. et al., 2003; Paszek et al., 2005). In addition, it was reported that increased ECM stiffness could also activate the SRC-YAP-MYL9/MYL2 axis in CAFs to maintain the CAF phenotype. A positive feedback loop is established between CAF function and ECM stiffness, leading the stiff tumor stroma to become even stiffer and more favorable for cancer cell invasion (Calvo et al., 2013).

**Table 3:** Integrins and their ECM partners.

ECM Protein	Interacting Integrins	References
Collagen	α1β1, α2β1, α10β1, α11β1	Leitinger, 2011
Fibronectin	α4β1, α5β1, α8β1, αIIbβ1, αvβ3, αvβ6, αvβ8	Pankov and Yamada, 2002; Danen et al., 2005
Tenascin-C	α9β1, α8β1, αvβ1, αvβ6	Tucker and Chiquet-Ehrismann, 2015
Laminin	α3β1, α6β1, α7β1, α6β4	Belkin and Stepp, 2000; Yamada and Sekiguchi, 2015

The “inside-out” signaling mode refers to the regulation of integrin-ECM interactions by intracellular signals. CAFs respond to tissue tension and exert their ECM remodeling and assembly abilities to further increase the stiffness of the stroma. The “inside–out” signaling mode is normally triggered by the binding of intracellular molecules, such as talin or kindlin, to the tails of integrins, leading to an increased affinity for extracellular ligands and enhanced ECM signaling (Shattil et al., 2010). For example, CAFs exert contractile forces and mediate extracellular FN1 assembly mainly via integrin αvβ3, leading to increased FN1 fibrillogenesis and ultimately contributing to tumor invasion (Attieh et al., 2017). Similarly, FN1 production and assembly were also observed in CAFs in prostate cancer. Erdogan et al. (2017) reported that CAFs produce an FN1-rich ECM with anisotropic fiber orientation as compared with normal fibroblasts and regulate cancer cell migration. In their study, CAFs remodel the FN1-rich ECM via the non-muscle myosin II (NMII)-α5β1 integrin axis.

## Direct CAF-Cancer Cell Contact

**FIGURE 3:**
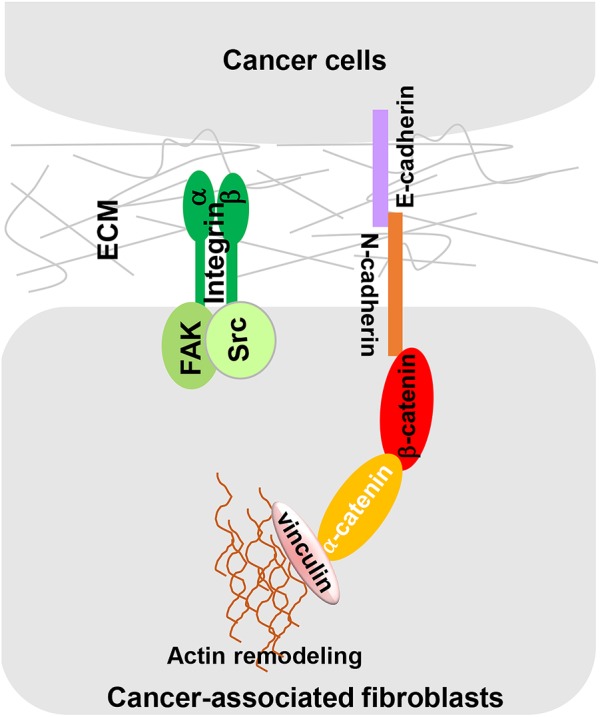
A heterotypic E-cadherin/N-cadherin complex mediates CAF-SCC cell contact. As reported by Labernadie et al. (2017) intercellular physical force is transmitted between SCCs and CAFs by a heterophilic adhesion complex involving N-cadherin at the CAF membrane and E-cadherin at the cancer cell membrane. This heterotypic CAF-cancer cell interaction triggers β-catenin recruitment, α-catenin/vinculin interaction, and actin remodeling, allowing CAFs to exert an intercellular physical force on cancer cells and promote cooperative tumor invasion.

Cancer-associated fibroblast-dependent tumor-promoting roles have long been attributed to the CAF secretome, but there is no denying that direct cell-cell contact also plays an important role in CAF-mediated cancer cell migration and invasion. Labernadie et al. (2017) discovered a heterotypic E-cadherin/N-cadherin adhesion complex between CAF and SCC cells. As shown in Figure 3, CAFs migrate through the ECM via integrin-mediated cytoskeleton remodeling and actomyosin reassembly while dragging tumor cells through CAF-cancer cell interaction via this heterotypic cadherin complex. The intercellular physical force between cancer cells and CAFs promote cooperative tumor invasion by triggering β-catenin recruitment, β-catenin/vinculin interaction and actin remodeling in both cell types.

In NSCLC, CAFs could potently enhance the motility of NSCLC cells through direct cell-cell contact via the hedgehog signaling pathway. Two co-culture systems (direct co-culture and indirect co-culture) were utilized to differentiate whether the motility-promoting effect is mediated by paracrine factors or cell-cell contact. Interestingly, increased tumor cell migration was only shown in a direct co-culture system, suggesting that CAF-promoted NSCLC cell migration is mediated by direct cell-cell contact (Choe et al., 2013). PDPN is a transmembrane glycoprotein that is known to be correlated with poor patient prognosis in lung adenocarcinoma (Ito et al., 2012). In an *in vitro* 3D collagen invasion model, PDPN-positive (PDPN+) CAFs accelerated lung tumor cell invasion into the collagen matrix. Ablation of PDPN reduced the invasive behavior of both CAFs and lung tumor cells. Because PDPN+ CAFs were observed to display high RHOA activity, RHO Kinase (ROCK) inhibitors were used to treat CAFs before co-culturing with lung tumor cells. ROCK inhibition suppressed PDPN-induced tumor cell migration, highlighting the role of the RHOA/ROCK axis in CAF-dependent tumor invasion (Neri et al., 2015).

## Indirect CAF-Cancer Cell Interactions

Paracrine signaling between CAFs and cancer cells represents another well-studied mode of interaction between the two cell types that shapes the TME and promotes tumor growth. Hepatocyte growth factor (HGF) is a paracrine growth factor known to contribute to cancer progression. In cancer cells, HGF activates downstream RAS/MAPK and PI3K signaling pathways by binding to its receptor MET (Organ and Tsao, 2011). Cytokine antibody arrays suggested that HGF was the most significantly upregulated secreted factor in CAFs in breast cancer when compared to normal fibroblasts, which is positively correlated to their pro-tumorigenic ability to promote breast tumorigenesis in mice (Tyan et al., 2011). Similarly, the tumor-promoting functions of CAF-derived HGF were also observed in gastric cancer. By ablating HGF expression *in vivo*, CAFs failed to promote tumor growth in nude mice (Wu et al., 2013). Interestingly, CAF-derived HGF is also sufficient to induce RAF inhibitor resistance via the binding of its receptor MET and reactivation of the MAPK and PI3K/AKT signaling pathways in melanoma cells. 50 nM of recombinant HGF induced strong drug resistance to a BRAF inhibitor, vemurafenib, in several melanoma cell lines (Straussman et al., 2012).

CXCL12, also known as stromal cell-derived factor 1 (SDF1), is an important regulator in cancer initiation, angiogenesis, and metastasis (Orimo et al., 2005; Sugihara et al., 2015; Teng et al., 2016). In addition, CXCL12 was shown to induce angiogenesis by recruiting endothelial progenitor cells (EPCs) in breast cancer, thereby providing sufficient nutrients to fuel tumor growth and metastasis. Furthermore, after mice bearing breast cancer were treated with antibodies targeting CXCL12, reduced tumor volume and cell number were observed (Orimo et al., 2005). It was reported that CAF-derived CXCL12 activated TGF-β-regulated C-X-C chemokine receptor type 4 (CXCR4) expression in human prostatic epithelial BPH-1 cells to induce tumorigenesis. The CAF-conditioned medium was sufficient to induce CXCR4-AKT activation in BPH-1 cells *in vitro*. *In vivo* tumor grafting experiments also supported this claim. CXCR4-deficient prostate tumors were significantly smaller and less invasive as compared to control tumors, confirming the role of the CXCL12-CXCR4 axis in initiating tumor formation (Ao et al., 2007). The EMT process represents a pivotal mechanism used by cancer cells for migration and invasion. It was shown *in vitro* that CAF-derived CXCL12 functions as an important EMT inducer in breast cancer cells by regulating the Wnt/β-catenin signaling pathway (Shan et al., 2015). TGF-β is another multifunctional cytokine that is well-known for its role in inducing the EMT process. CAF-derived TGF-β1 promoted the aggressive phenotypes of breast cancer cells by inducing EMT through the activation of TGF-β/SMAD signaling. The EMT phenotype was reversed in the cells after the addition of TGF-β1 neutralizing antibody (Yu et al., 2014).

## Therapeutic Perspectives: Targeting the ECM Microenvironment

Despite the growing enthusiasm for the development of CAF-targeting therapies, targeting CAFs has been challenging and lacks real and meaningful progress. One interesting example is FAP. Murine anti-FAP antibody F19 showed a significant tumor-inhibitory effect in xenograft models of lung, pancreas, and head and neck cancers with no obvious signs of toxicity (Ostermann et al., 2008). Because of promising pre-clinical data, a humanized version of murine anti-FAP antibody, sibrotuzumab, has been designed and tested in a phase I clinical trial and was determined to be safe and tolerable (Scott et al., 2003). However, in the phase II study in metastatic colorectal cancer, sibrotuzumab showed no therapeutic benefits (Hofheinz et al., 2003). Therefore, instead of targeting a specific subset of CAFs or CAFs in general, identifying the exact mechanisms that CAFs use to support cancer cells may help to develop better therapeutic strategies, e.g., based on CAF autophagy (Zhang et al., 2018), or based on the specific ECM proteins that are produced by CAFs.

## Targeting ECM Proteins

Humanized anti-collagen antibodies and ECM inhibitors have emerged as promising agents for cancer therapy (de Jonge et al., 2006; Koon et al., 2011). Halofuginone is an inhibitor of collagen I and was shown having anti-tumor activities in mouse models of prostate cancer (Gavish et al., 2002), pancreatic cancer (Spector et al., 2010) and lung cancer (Taras et al., 2006). D93/TRC093 is a humanized monoclonal antibody that specifically binds the HU177 cryptic collagen epitope within the tumor ECM with potential antiangiogenic and antitumor activities (Cretu et al., 2007; Caron et al., 2016). In the study conducted by Caron et al. (2016) D93/TRC093 was found to restrict the accumulation of α-SMA+ fibroblasts, which could be explained by the inhibition of integrin α_10_β_1_-mediated fibroblast adhesion and migration on denatured collagen. In a phase I clinical study, TRC093 was shown to be well-tolerated and had tumor-inhibitory effects as monotherapy and in combination with bevacizumab in 19 patients carrying different types of solid tumors (Robert et al., 2010).

Conjugating a monoclonal antibody with a cell-killing agent is a new approach to develop novel targeted anti-cancer agents. In the past two decades, FN1-targeting antibodies have been designed and tested in different models. L19 is a monoclonal antibody known to target the ED-B domain of FN1. By attaching anti-angiogenesis drugs to L19, the fusion protein was demonstrated to exhibit strong anti-tumor effects in animal models carrying different tumors, including teratocarcinoma, colon adenocarcinoma and sarcoma (Birchler et al., 1999). Interleukin-2 (IL-2) is a cytokine factor and an important player in anticancer immunity. However, the cardiovascular toxicity of IL-2 remains a major clinical issue. To overcome this problem, a new strategy was designed by fusing IL-2 with L19 so that IL-2 can be precisely targeted to the tumor site, resulting in reduced side effects. This drug conjugate exerted strong immune-stimulatory effects and inhibited tumor growth in stage III melanoma patients (Danielli et al., 2015). Currently, L19-IL-2 in combination with L19-TNF is in a phase III clinical trial to evaluate its efficacy against advanced melanoma (ClinicalTrials.gov Identifier: NCT03567889). Similarly, TNC-targeting antibodies have also been conjugated with IL-2, and have shown some preliminary signs of anti-tumor activity in advanced solid tumors and metastatic breast cancer (Catania et al., 2015). Navitoclax (ABT-263) is a small molecule that was shown to have the ability to induce apoptosis in myofibroblasts (Lagares et al., 2017). Consequently, Navitoclax could be used to target CAFs in solid tumor. Navitoclax-loaded nanoliposome was modified with peptide FH (FH-SSL-Nav), which specifically binds to TNC, to precisely eradicate CAFs at the tumor site. Using a xenograft mouse model of hepatocellular cancer, FH-SSL-Nav was shown to have the ability to deplete CAFs and inhibit tumor growth (Chen et al., 2016). In January 2017, the National Cancer Institute (NCI) approved a phase Ib/II trial study to evaluate the side effects and best dose of the combination of MEK inhibitor Trametinib and Navitoclax in treating patients with advanced or metastatic solid tumors (ClinicalTrials.gov Identifier: NCT02079740).

## Targeting ECM Remodeling Enzymes

Extracellular matrix remodeling plays an essential role in CAF-mediated desmoplastic reactions, which cannot be achieved without LOX-induced ECM crosslinking. LOX inhibitors have emerged as potential alternatives to target the desmoplastic TME and improve drug delivery efficacy. In an *in vitro* 3D spheroid model using four different mouse tumor cell lines, including Lewis lung carcinoma cell line (LLC), a fibrosarcoma cell line (MT6) and two breast carcinoma cell lines (4T1, EMT6), LOX inhibition significantly improved the diffusion of doxorubicin (Schutze et al., 2015). Blocking LOX family oxidases *in vitro* or *in vivo* has shown potent anti-tumor activities in breast and pancreatic cancer (Park et al., 2016; Chang et al., 2017). Nevertheless, caution should still be taken when considering using LOX inhibitors. In a rat model of prostate cancer, LOX inhibition seems to have context-dependent effects during different stages of tumor progression. Before tumor formation, LOX inhibitors showed strong tumor-inhibiting capacity. To the contrary, after prostate tumors were established, LOX inhibition did not affect or decrease tumor growth (Nilsson et al., 2016). In recent clinical trials, simtuzumab, a monoclonal antibody against LOXL2, failed to produce improved anti-tumor benefits when given in combination with other anti-cancer drugs, including 5-fluorouracil, leucovorin, irinotecan (FOLFIRI) and gemcitabine (Benson et al., 2017; Hecht et al., 2017).

Many MMPs have been known to be notorious for their roles in promoting cancer progression. As a result, more than 50 MMP inhibitors were investigated in clinical trials. In a pre-clinical study, an anti-MMP9 monoclonal antibody GS-5745 successfully inhibited tumor growth and reduced tumor metastasis in mice bearing colorectal tumors (Marshall et al., 2015). Nevertheless, despite exciting preclinical data, none of these MMP inhibitors displayed anti-tumor effects in clinical trials. Although there are many explanations for these failures, such as bad clinical trial design, poor oral bioavailability, and inadequate cancer stages (Vandenbroucke and Libert, 2014), one potential reason responsible for the failures of these MMP inhibitors might be the obscurity of the roles and functions of MMPs in the ECM microenvironment. In addition, the use of broad-spectrum MMP inhibitors also suppresses potential tumor-inhibiting MMPs. Therefore, although MMPs are attractive therapeutic targets, more research is needed to unravel the roles of different MMPs in different cancer types and/or during various cancer stages. Furthermore, more efforts are required to develop more specific and selective MMP inhibitors to avoid potential side effects.

## Targeting CAF-Derived Molecular Signals

Cancer-associated fibroblast-mediated paracrine signaling has also been envisioned as a potential target in cancer treatment. In a recent phase I–II study on myeloid leukemia, plerixafor, a CXCR4 inhibitor, resulted in improved recovery rate when given in combination with a FLAG-Ida regime (fludarabine, idarubicin, cytarabine, and G-CSF) (Martinez-Cuadron et al., 2018). To block TGF-β activity, TGF-β inhibitors and monoclonal antibodies have been designed and tested in clinical trials. Galunisertib, a TGF-β receptor kinase inhibitor, however, showed highly context-dependent tumor-inhibitory effects. While it showed promising clinical responses in neuroblastoma patients (Tran et al., 2017), galunisertib had no significant therapeutic effect in a phase II clinical study in recurrent glioblastoma patients (Brandes et al., 2016). The monoclonal antibody fresolimumab (GC1008), which is capable of neutralizing all human isoforms of TGF-β, has also been investigated in advanced malignant melanoma and renal cell carcinoma and showed early stage anti-tumor effects with no dose-limiting toxicity in a phase I clinical study (Morris et al., 2014). In 2017, several clinical trials investigating an anti-HGF antibody, rilotumumab, were published. In one clinical trial, improved antitumor activities of rilotumumab in combination with cisplatin and capecitabine were shown in patients with MET-positive advanced gastric or gastroesophageal junction cancer (Doi et al., 2017). The combined use of rilotumumab with erlotinib (an EGFR receptor inhibitor) also showed successes in treating advanced NSCLC (Tarhini et al., 2017). However, in another clinical trial on small-cell lung cancer patients, no significant clinical benefit of rilotumumab in combination with platinum-based chemotherapy was observed (Glisson et al., 2017). Similarly, in a phase III clinical study, the treatment utilizing rilotumumab plus epirubicin, cisplatin, and capecitabine as a first-line therapy on gastric or gastro-oesophageal junction cancer patients was unsuccessful (Catenacci et al., 2017). Taken together, targeting CAF-induced paracrine signaling appears to be spatial-temporal and case-dependent.

## Conclusion

It is an astonishing feat of the tumor cells to abandon the basic rules of tissue homeostasis and to grow uncontrollably. Unfortunately, as we have learned from many modern targeted therapies, a simple approach to eliminate tumor “seed” is generally condemned to failure. It is becoming clear that the TME is actively involved in tumor initiation, progression, metastasis and the development of drug resistance. However, only after gaining enhanced knowledge about the TME, including the heterogeneous nature and complexity of CAF populations, a multiplex approach targeting CAFs and the ECM will naturally come by and provide desired clinical benefits.

## Author Contributions

All authors listed have made a substantial, direct and intellectual contribution to the work, and approved it for publication.

## Conflict of Interest Statement

The authors declare that the research was conducted in the absence of any commercial or financial relationships that could be construed as a potential conflict of interest.

## References

[B1] Ao M. Franco O. E. Park D. Raman D. Williams K. Hayward S. W. (2007). Cross-talk between paracrine-acting cytokine and chemokine pathways promotes malignancy in benign human prostatic epithelium. *Cancer Res.* 67 4244–4253. 10.1158/0008-5472.CAN-06-3946 17483336

[B2] Ashley S. L. Wilke C. A. Kim K. K. Moore B. B. (2017). Periostin regulates fibrocyte function to promote myofibroblast differentiation and lung fibrosis. *Mucosal Immunol.* 10 341–351. 10.1038/mi.2016.61 27435108 PMC5250615

[B3] Attieh Y. Clark A. G. Grass C. Richon S. Pocard M. Mariani P. (2017). Cancer-associated fibroblasts lead tumor invasion through integrin-beta3-dependent fibronectin assembly. *J. Cell Biol.* 216 3509–3520. 10.1083/jcb.201702033 28931556 PMC5674886

[B4] Barczyk M. Carracedo S. Gullberg D. (2010). Integrins. *Cell Tissue Res.* 339 269–280. 10.1007/s00441-009-0834-6 19693543 PMC2784866

[B5] Barry-Hamilton V. Spangler R. Marshall D. McCauley S. Rodriguez H. M. Oyasu M. (2010). Allosteric inhibition of lysyl oxidase-like-2 impedes the development of a pathologic microenvironment. *Nat. Med.* 16 1009–1017. 10.1038/nm.2208 20818376

[B6] Bates A. L. Pickup M. W. Hallett M. A. Dozier E. A. Thomas S. Fingleton B. (2015). Stromal matrix metalloproteinase 2 regulates collagen expression and promotes the outgrowth of experimental metastases. *J. Pathol.* 235 773–783. 10.1002/path.4493 25469981 PMC4357558

[B7] Bauer M. Su G. Casper C. He R. Rehrauer W. Friedl A. (2010). Heterogeneity of gene expression in stromal fibroblasts of human breast carcinomas and normal breast. *Oncogene* 29 1732–1740. 10.1038/onc.2009.463 20062080 PMC2845730

[B8] Baum J. Duffy H. S. (2011). Fibroblasts and myofibroblasts: what are we talking about? *J. Cardiovasc. Pharmacol.* 57 376–379. 10.1097/FJC.0b013e3182116e39 21297493 PMC3077448

[B9] Belkin A. M. Stepp M. A. (2000). Integrins as receptors for laminins. *Microsc. Res. Tech.* 51 280–301. 11054877 10.1002/1097-0029(20001101)51:3<280::AID-JEMT7>3.0.CO;2-O

[B10] Bellomo C. Caja L. Moustakas A. (2016). Transforming growth factor beta as regulator of cancer stemness and metastasis. *Br. J. Cancer* 115 761–769. 10.1038/bjc.2016.255 27537386 PMC5046208

[B11] Benito-Jardon M. Klapproth S. Gimeno L. I. Petzold T. Bharadwaj M. Muller D. J. (2017). The fibronectin synergy site re-enforces cell adhesion and mediates a crosstalk between integrin classes. *eLife* 6:e22264. 10.7554/eLife.22264 28092265 PMC5279944

[B12] Benson A. B. III Wainberg Z. A. Hecht J. R. Vyushkov D. Dong H. Bendell J. (2017). A phase II randomized, double-blind, placebo-controlled study of simtuzumab or placebo in combination with gemcitabine for the first-line treatment of pancreatic adenocarcinoma. *Oncologist* 22 241–e15. 10.1634/theoncologist.2017-0024 28246206 PMC5344644

[B13] Biffi G. Oni T. E. Spielman B. Hao Y. Elyada E. Park Y. (2019). IL1-induced JAK/STAT signaling is antagonized by TGFbeta to shape CAF heterogeneity in pancreatic ductal adenocarcinoma. *Cancer Discov.* 9 282–301. 10.1158/2159-8290.CD-18-0710 30366930 PMC6368881

[B14] Birchler M. Viti F. Zardi L. Spiess B. Neri D. (1999). Selective targeting and photocoagulation of ocular angiogenesis mediated by a phage-derived human antibody fragment. *Nat. Biotechnol.* 17 984–988. 10.1038/13679 10504699

[B15] Botti G. Cerrone M. Scognamiglio G. Anniciello A. Ascierto P. A. Cantile M. (2013). Microenvironment and tumor progression of melanoma: new therapeutic prospectives. *J. Immunotoxicol.* 10 235–252. 10.3109/1547691X.2012.723767 23036080

[B16] Brandes A. A. Carpentier A. F. Kesari S. Sepulveda-Sanchez J. M. Wheeler H. R. Chinot O. (2016). A phase II randomized study of galunisertib monotherapy or galunisertib plus lomustine compared with lomustine monotherapy in patients with recurrent glioblastoma. *Neuro Oncol.* 18 1146–1156. 10.1093/neuonc/now009 26902851 PMC4933481

[B17] Brellier F. Hostettler K. Hotz H. R. Ozcakir C. Cologlu S. A. Togbe D. (2011). Tenascin-C triggers fibrin accumulation by downregulation of tissue plasminogen activator. *FEBS Lett.* 585 913–920. 10.1016/j.febslet.2011.02.023 21354146

[B18] Cai J. Du S. Wang H. Xin B. Wang J. Shen W. (2017). Tenascin-C induces migration and invasion through JNK/c-Jun signalling in pancreatic cancer. *Oncotarget* 8 74406–74422. 10.18632/oncotarget.20160 29088796 PMC5650351

[B19] Calvo F. Ege N. Grande-Garcia A. Hooper S. Jenkins R. P. Chaudhry S. I. (2013). Mechanotransduction and YAP-dependent matrix remodelling is required for the generation and maintenance of cancer-associated fibroblasts. *Nat. Cell Biol.* 15 637–646. 10.1038/ncb2756 23708000 PMC3836234

[B20] Caron J. M. Ames J. J. Contois L. Liebes L. Friesel R. Muggia F. (2016). Inhibition of ovarian tumor growth by targeting the HU177 cryptic collagen epitope. *Am. J. Pathol.* 186 1649–1661. 10.1016/j.ajpath.2016.01.015 27216148 PMC4901133

[B21] Carracedo S. Lu N. Popova S. N. Jonsson R. Eckes B. Gullberg D. (2010). The fibroblast integrin alpha11beta1 is induced in a mechanosensitive manner involving activin A and regulates myofibroblast differentiation. *J. Biol. Chem.* 285 10434–10445. 10.1074/jbc.M109.078766 20129924 PMC2856250

[B22] Catania C. Maur M. Berardi R. Rocca A. Giacomo A. M. Spitaleri G. (2015). The tumor-targeting immunocytokine F16-IL2 in combination with doxorubicin: dose escalation in patients with advanced solid tumors and expansion into patients with metastatic breast cancer. *Cell Adh. Migr.* 9 14–21. 10.4161/19336918.2014.983785 25562532 PMC4422815

[B23] Catenacci D. V. T. Tebbutt N. C. Davidenko I. Murad A. M. Al-Batran S. E. Ilson D. H. (2017). Rilotumumab plus epirubicin, cisplatin, and capecitabine as first-line therapy in advanced MET-positive gastric or gastro-oesophageal junction cancer (RILOMET-1): a randomised, double-blind, placebo-controlled, phase 3 trial. *Lancet Oncol.* 18 1467–1482. 10.1016/S1470-2045(17)30566-1 28958504 PMC5898242

[B24] Chang J. Lucas M. C. Leonte L. E. Garcia-Montolio M. Singh L. B. Findlay A. D. (2017). Pre-clinical evaluation of small molecule LOXL2 inhibitors in breast cancer. *Oncotarget* 8 26066–26078. 10.18632/oncotarget.15257 28199967 PMC5432238

[B25] Chen B. Wang Z. Sun J. Song Q. He B. Zhang H. (2016). A tenascin C targeted nanoliposome with navitoclax for specifically eradicating of cancer-associated fibroblasts. *Nanomedicine* 12 131–141. 10.1016/j.nano.2015.10.001 26518604

[B26] Chen S. Z. Yao H. Q. Zhu S. Z. Li Q. Y. Guo G. H. Yu J. (2015). Expression levels of matrix metalloproteinase-9 in human gastric carcinoma. *Oncol. Lett.* 9 915–919. 10.3892/ol.2014.2768 25621068 PMC4301519

[B27] Chen Y. Wang X. Chen G. Dong C. Zhang D. (2015). The impact of matrix metalloproteinase 2 on prognosis and clinicopathology of breast cancer patients: a systematic meta-analysis. *PLoS One* 10:e0121404. 10.1371/journal.pone.0121404 25816052 PMC4376789

[B28] Choe C. Shin Y. S. Kim S. H. Jeon M. J. Choi S. J. Lee J. (2013). Tumor-stromal interactions with direct cell contacts enhance motility of non-small cell lung cancer cells through the hedgehog signaling pathway. *Anticancer Res.* 33 3715–3723. 24023301

[B29] Costa A. Kieffer Y. Scholer-Dahirel A. Pelon F. Bourachot B. Cardon M. (2018). Fibroblast heterogeneity and immunosuppressive environment in human breast cancer. *Cancer Cell* 33 463–479.e10. 10.1016/j.ccell.2018.01.011 29455927

[B30] Cretu A. Roth J. M. Caunt M. Akalu A. Policarpio D. Formenti S. (2007). Disruption of endothelial cell interactions with the novel HU177 cryptic collagen epitope inhibits angiogenesis. *Clin. Cancer Res.* 13 3068–3078. 10.1158/1078-0432.CCR-06-2342 17505010

[B31] Danen E. H. van Rheenen J. Franken W. Huveneers S. Sonneveld P. Jalink K. (2005). Integrins control motile strategy through a Rho-cofilin pathway. *J. Cell Biol.* 169 515–526. 10.1083/jcb.200412081 15866889 PMC2171933

[B32] Danielli R. Patuzzo R. Di Giacomo A. M. Gallino G. Maurichi A. Di Florio A. (2015). Intralesional administration of L19-IL2/L19-TNF in stage III or stage IVM1a melanoma patients: results of a phase II study. *Cancer Immunol. Immunother.* 64 999–1009. 10.1007/s00262-015-1704-6 25971540 PMC11028725

[B33] Darby I. A. Laverdet B. Bonte F. Desmouliere A. (2014). Fibroblasts and myofibroblasts in wound healing. *Clin. Cosmet. Investig. Dermatol.* 7 301–311. 10.2147/CCID.S50046 25395868 PMC4226391

[B34] de Groot A. E. Roy S. Brown J. S. Pienta K. J. Amend S. R. (2017). Revisiting seed and soil: examining the primary tumor and cancer cell foraging in metastasis. *Mol. Cancer Res.* 15 361–370. 10.1158/1541-7786.MCR-16-0436 28209759 PMC5380470

[B35] de Jonge M. J. Dumez H. Verweij J. Yarkoni S. Snyder D. Lacombe D. (2006). Phase I and pharmacokinetic study of halofuginone, an oral quinazolinone derivative in patients with advanced solid tumours. *Eur. J. Cancer* 42 1768–1774. 10.1016/j.ejca.2005.12.027 16815702

[B36] De Palma M. Biziato D. Petrova T. V. (2017). Microenvironmental regulation of tumour angiogenesis. *Nat. Rev. Cancer* 17 457–474. 10.1038/nrc.2017.51 28706266

[B37] Desmouliere A. Redard M. Darby I. Gabbiani G. (1995). Apoptosis mediates the decrease in cellularity during the transition between granulation tissue and scar. *Am. J. Pathol.* 146 56–66. 7856739 PMC1870783

[B38] Doi T. Yamaguchi K. Komatsu Y. Muro K. Nishina T. Nakajima T. E. (2017). A Phase 1/1b tolerability study of rilotumumab alone or in combination with cisplatin and capecitabine in Japanese patients with gastric cancer. *Jpn. J. Clin. Oncol.* 47 1002–1009. 10.1093/jjco/hyx114 28973403

[B39] Dvorak H. F. (1986). Tumors: wounds that do not heal. Similarities between tumor stroma generation and wound healing. *N. Engl. J. Med.* 315 1650–1659. 10.1056/NEJM198612253152606 3537791

[B40] Erdogan B. Ao M. White L. M. Means A. L. Brewer B. M. Yang L. (2017). Cancer-associated fibroblasts promote directional cancer cell migration by aligning fibronectin. *J. Cell Biol.* 216 3799–3816. 10.1083/jcb.201704053 29021221 PMC5674895

[B41] Faouzi S. Le Bail B. Neaud V. Boussarie L. Saric J. Bioulac-Sage P. (1999). Myofibroblasts are responsible for collagen synthesis in the stroma of human hepatocellular carcinoma: an in vivo and in vitro study. *J. Hepatol.* 30 275–284. 10068108 10.1016/s0168-8278(99)80074-9

[B42] Flavell R. A. Sanjabi S. Wrzesinski S. H. Licona-Limon P. (2010). The polarization of immune cells in the tumour environment by TGFbeta. *Nat. Rev. Immunol.* 10 554–567. 10.1038/nri2808 20616810 PMC3885992

[B43] Foster D. S. Jones R. E. Ransom R. C. Longaker M. T. Norton J. A. (2018). The evolving relationship of wound healing and tumor stroma. *JCI Insight* 3:99911. 10.1172/jci.insight.99911 30232274 PMC6237224

[B44] Fu Z. Xu S. Xu Y. Ma J. Li J. Xu P. (2015). The expression of tumor-derived and stromal-derived matrix metalloproteinase 2 predicted prognosis of ovarian cancer. *Int. J. Gynecol. Cancer* 25 356–362. 10.1097/IGC.0000000000000386 25695542 PMC4340603

[B45] Fukino K. Shen L. Matsumoto S. Morrison C. D. Mutter G. L. Eng C. (2004). Combined total genome loss of heterozygosity scan of breast cancer stroma and epithelium reveals multiplicity of stromal targets. *Cancer Res.* 64 7231–7236. 10.1158/0008-5472.CAN-04-2866 15492239

[B46] Fullar A. Dudas J. Olah L. Hollosi P. Papp Z. Sobel G. (2015). Remodeling of extracellular matrix by normal and tumor-associated fibroblasts promotes cervical cancer progression. *BMC Cancer* 15:256. 10.1186/s12885-015-1272-3 25885552 PMC4409756

[B47] Gao A. E. Sullivan K. E. Black L. D. III (2016). Lysyl oxidase expression in cardiac fibroblasts is regulated by alpha2beta1 integrin interactions with the cellular microenvironment. *Biochem. Biophys. Res. Commun.* 475 70–75. 10.1016/j.bbrc.2016.05.037 27169768

[B48] Gavish Z. Pinthus J. H. Barak V. Ramon J. Nagler A. Eshhar Z. (2002). Growth inhibition of prostate cancer xenografts by halofuginone. *Prostate* 51 73–83. 10.1002/pros.10059 11948962

[B49] Glisson B. Besse B. Dols M. C. Dubey S. Schupp M. Jain R. (2017). A randomized, placebo-controlled, phase 1b/2 study of rilotumumab or ganitumab in combination with platinum-based chemotherapy as first-line treatment for extensive-stage small-cell lung cancer. *Clin. Lung Cancer* 18 615–625.e8. 10.1016/j.cllc.2017.05.007 28601388

[B50] Gong L. Wu D. Zou J. Chen J. Chen L. Chen Y. (2016). Prognostic impact of serum and tissue MMP-9 in non-small cell lung cancer: a systematic review and meta-analysis. *Oncotarget* 7 18458–18468. 10.18632/oncotarget.7607 26918342 PMC4951301

[B51] Gozdzialska A. Wojas-Pelc A. Drag J. Brzewski P. Jaskiewicz J. Pastuszczak M. (2016). Expression of metalloproteinases (MMP-2 and MMP-9) in basal-cell carcinoma. *Mol. Biol. Rep.* 43 1027–1033. 10.1007/s11033-016-4040-9 27406386 PMC5025502

[B52] Groblewska M. Mroczko B. Gryko M. Pryczynicz A. Guzinska-Ustymowicz K. Kedra B. (2014). Serum levels and tissue expression of matrix metalloproteinase 2 (MMP-2) and tissue inhibitor of metalloproteinases 2 (TIMP-2) in colorectal cancer patients. *Tumour Biol.* 35 3793–3802. 10.1007/s13277-013-1502-8 24395652 PMC3980035

[B53] Harper R. A. Grove G. (1979). Human skin fibroblasts derived from papillary and reticular dermis: differences in growth potential in vitro. *Science* 204 526–527. 10.1126/science.432659 432659

[B54] Hecht J. R. Benson A. B. III Vyushkov D. Yang Y. Bendell J. Verma U. (2017). A phase II, randomized, double-blind, placebo-controlled study of simtuzumab in combination with FOLFIRI for the second-line treatment of metastatic KRAS mutant colorectal adenocarcinoma. *Oncologist* 22 243–e23. 10.1634/theoncologist.2016-0479 28246207 PMC5344646

[B55] Hikage F. Atkins S. Kahana A. Smith T. J. Chun T. H. (2019). HIF2A-LOX pathway promotes fibrotic tissue remodeling in thyroid-associated orbitopathy. *Endocrinology* 160 20–35. 10.1210/en.2018-00272 30388216 PMC6293089

[B56] Hinz B. Phan S. H. Thannickal V. J. Galli A. Bochaton-Piallat M. L. Gabbiani G. (2007). The myofibroblast: one function, multiple origins. *Am. J. Pathol.* 170 1807–1816. 10.2353/ajpath.2007.070112 17525249 PMC1899462

[B57] Hofheinz R. D. al-Batran S. E. Hartmann F. Hartung G. Jager D. Renner C. (2003). Stromal antigen targeting by a humanised monoclonal antibody: an early phase II trial of sibrotuzumab in patients with metastatic colorectal cancer. *Onkologie* 26 44–48. 10.1159/000069863 12624517

[B58] Hsia L. T. Ashley N. Ouaret D. Wang L. M. Wilding J. Bodmer W. F. (2016). Myofibroblasts are distinguished from activated skin fibroblasts by the expression of AOC3 and other associated markers. *Proc. Natl. Acad. Sci. U.S.A.* 113 E2162–E2171. 10.1073/pnas.1603534113 27036009 PMC4839407

[B59] Ito M. Ishii G. Nagai K. Maeda R. Nakano Y. Ochiai A. (2012). Prognostic impact of cancer-associated stromal cells in patients with stage I lung adenocarcinoma. *Chest* 142 151–158. 10.1378/chest.11-2458 22302300

[B60] Iwaisako K. Brenner D. A. Kisseleva T. (2012). What’s new in liver fibrosis? The origin of myofibroblasts in liver fibrosis. *J. Gastroenterol. Hepatol.* 27(Suppl. 2) 65–68. 10.1111/j.1440-1746.2011.07002.x 22320919 PMC4841268

[B61] Iyer R. P. Patterson N. L. Fields G. B. Lindsey M. L. (2012). The history of matrix metalloproteinases: milestones, myths, and misperceptions. *Am. J. Physiol. Heart Circ. Physiol.* 303 H919–H930. 10.1152/ajpheart.00577.2012 22904159 PMC3469639

[B62] Jakubowska K. Pryczynicz A. Januszewska J. Sidorkiewicz I. Kemona A. Niewinski A. (2016). Expressions of matrix metalloproteinases 2,7, and 9 in carcinogenesis of pancreatic ductal adenocarcinoma. *Dis. Markers* 2016:9895721. 10.1155/2016/9895721 27429508 PMC4939209

[B63] Janson D. G. Saintigny G. van Adrichem A. Mahe C. El Ghalbzouri A. (2012). Different gene expression patterns in human papillary and reticular fibroblasts. *J. Invest. Dermatol.* 132 2565–2572. 10.1038/jid.2012.192 22696053

[B64] Kamyab-Hesari K. Mohtasham N. Aghazadeh N. Biglarian M. Memar B. Kadeh H. (2014). The expression of MMP-2 and Ki-67 in head and neck melanoma, and their correlation with clinic-pathologic indices. *J. Cancer Res. Ther.* 10 696–700. 10.4103/0973-1482.138122 25313763

[B65] Kanisicak O. Khalil H. Ivey M. J. Karch J. Maliken B. D. Correll R. N. (2016). Genetic lineage tracing defines myofibroblast origin and function in the injured heart. *Nat. Commun.* 7:12260. 10.1038/ncomms12260 27447449 PMC5512625

[B66] Kasashima H. Yashiro M. Kinoshita H. Fukuoka T. Morisaki T. Masuda G. (2014). Lysyl oxidase-like 2 (LOXL2) from stromal fibroblasts stimulates the progression of gastric cancer. *Cancer Lett.* 354 438–446. 10.1016/j.canlet.2014.08.014 25128648

[B67] Koon H. B. Fingleton B. Lee J. Y. Geyer J. T. Cesarman E. Parise R. A. (2011). Phase II AIDS Malignancy Consortium trial of topical halofuginone in AIDS-related Kaposi sarcoma. *J. Acquir. Immune Defic. Syndr.* 56 64–68. 10.1097/QAI.0b013e3181fc0141 21068672 PMC3017346

[B68] Korosec A. Frech S. Gesslbauer B. Vierhapper M. Radtke C. Petzelbauer P. (2019). Lineage identity and location within the dermis determine the function of papillary and reticular fibroblasts in human skin. *J. Invest. Dermatol.* 139 342–351. 10.1016/j.jid.2018.07.033 30179601

[B69] Kumar P. Smith T. Raeman R. Chopyk D. M. Brink H. Liu Y. (2018). Periostin promotes liver fibrogenesis by activating lysyl oxidase in hepatic stellate cells. *J. Biol. Chem.* 293 12781–12792. 10.1074/jbc.RA117.001601 29941453 PMC6102155

[B70] Labernadie A. Kato T. Brugues A. Serra-Picamal X. Derzsi S. Arwert E. (2017). A mechanically active heterotypic E-cadherin/N-cadherin adhesion enables fibroblasts to drive cancer cell invasion. *Nat. Cell Biol.* 19 224–237. 10.1038/ncb3478 28218910 PMC5831988

[B71] Lagares D. Santos A. Grasberger P. E. Liu F. Probst C. K. Rahimi R. A. (2017). Targeted apoptosis of myofibroblasts with the BH3 mimetic ABT-263 reverses established fibrosis. *Sci. Transl. Med.* 9:eaal3765. 10.1126/scitranslmed.aal3765 29237758 PMC8520471

[B72] Lambrechts D. Wauters E. Boeckx B. Aibar S. Nittner D. Burton O. (2018). Phenotype molding of stromal cells in the lung tumor microenvironment. *Nat. Med.* 24 1277–1289. 10.1038/s41591-018-0096-5 29988129

[B73] LeBleu V. S. Kalluri R. (2018). A peek into cancer-associated fibroblasts: origins, functions and translational impact. *Dis. Model. Mech.* 11:dmm029447. 10.1242/dmm.029447 29686035 PMC5963854

[B74] Lee C. Y. Shim H. S. Lee S. Lee J. G. Kim D. J. Chung K. Y. (2015). Prognostic effect of matrix metalloproteinase-9 in patients with resected Non small cell lung cancer. *J. Cardiothorac. Surg.* 10:44. 10.1186/s13019-015-0248-3 25888323 PMC4379698

[B75] Leitinger B. (2011). Transmembrane collagen receptors. *Annu. Rev. Cell Dev. Biol.* 27 265–290. 10.1146/annurev-cellbio-092910-154013 21568710

[B76] Li H. Courtois E. T. Sengupta D. Tan Y. Chen K. H. Goh J. J. L. (2017). Reference component analysis of single-cell transcriptomes elucidates cellular heterogeneity in human colorectal tumors. *Nat. Genet.* 49 708–718. 10.1038/ng.3818 28319088

[B77] Li Q. Chen B. Cai J. Sun Y. Wang G. Li Y. (2016). Comparative analysis of matrix metalloproteinase family members reveals that MMP9 predicts survival and response to temozolomide in patients with primary glioblastoma. *PLoS One* 11:e0151815. 10.1371/journal.pone.0151815 27022952 PMC4811585

[B78] Li S. Shen X. Yang Z. Wu A. Tang Z. Li M. (2014). [Clinical significance of MMP2 overexpression in endometrial adenocarcinoma]. *Nan Fang Yi Ke Da Xue Xue Bao* 34 423–425. 24670463

[B79] Liu H. Y. Gu W. J. Wang C. Z. Ji X. J. Mu Y. M. (2016). Matrix metalloproteinase-9 and -2 and tissue inhibitor of matrix metalloproteinase-2 in invasive pituitary adenomas: a systematic review and meta-analysis of case-control trials. *Medicine* 95:e3904. 10.1097/MD.0000000000003904 27310993 PMC4998479

[B80] Marshall D. C. Lyman S. K. McCauley S. Kovalenko M. Spangler R. Liu C. (2015). Selective allosteric inhibition of MMP9 is efficacious in preclinical models of ulcerative colitis and colorectal cancer. *PLoS One* 10:e0127063. 10.1371/journal.pone.0127063 25961845 PMC4427291

[B81] Martinez-Cuadron D. Boluda B. Martinez P. Bergua J. Rodriguez-Veiga R. Esteve J. (2018). A phase I-II study of plerixafor in combination with fludarabine, idarubicin, cytarabine, and G-CSF (PLERIFLAG regimen) for the treatment of patients with the first early-relapsed or refractory acute myeloid leukemia. *Ann. Hematol.* 97 763–772. 10.1007/s00277-018-3229-5 29392425

[B82] Matthijs Blankesteijn W. (2015). Has the search for a marker of activated fibroblasts finally come to an end? *J. Mol. Cell Cardiol.* 88 120–123. 10.1016/j.yjmcc.2015.10.005 26454160

[B83] McAnulty R. J. (2007). Fibroblasts and myofibroblasts: their source, function and role in disease. *Int. J. Biochem. Cell Biol.* 39 666–671. 10.1016/j.biocel.2006.11.005 17196874

[B84] McAnulty R. J. Campa J. S. Cambrey A. D. Laurent G. J. (1991). The effect of transforming growth factor beta on rates of procollagen synthesis and degradation in vitro. *Biochim. Biophys. Acta* 1091 231–235. 1995081 10.1016/0167-4889(91)90066-7

[B85] Menzin A. W. Loret de Mola J. R. Bilker W. B. Wheeler J. E. Rubin S. C. (1998). Identification of oncofetal fibronectin in patients with advanced epithelial ovarian cancer: detection in ascitic fluid and localization to primary sites and metastatic implants. *Cancer* 82 152–158. 9428492 10.1002/(sici)1097-0142(19980101)82:1<152::aid-cncr19>3.0.co;2-1

[B86] Moore-Morris T. Guimaraes-Camboa N. Banerjee I. Zambon A. C. Kisseleva T. Velayoudon A. (2014). Resident fibroblast lineages mediate pressure overload-induced cardiac fibrosis. *J. Clin. Invest.* 124 2921–2934. 10.1172/JCI74783 24937432 PMC4071409

[B87] Mori L. Bellini A. Stacey M. A. Schmidt M. Mattoli S. (2005). Fibrocytes contribute to the myofibroblast population in wounded skin and originate from the bone marrow. *Exp. Cell Res.* 304 81–90. 10.1016/j.yexcr.2004.11.011 15707576

[B88] Morris J. C. Tan A. R. Olencki T. E. Shapiro G. I. Dezube B. J. Reiss M. (2014). Phase I study of GC1008 (fresolimumab): a human anti-transforming growth factor-beta (TGFbeta) monoclonal antibody in patients with advanced malignant melanoma or renal cell carcinoma. *PLoS One* 9:e90353. 10.1371/journal.pone.0090353 24618589 PMC3949712

[B89] Neri S. Ishii G. Hashimoto H. Kuwata T. Nagai K. Date H. (2015). Podoplanin-expressing cancer-associated fibroblasts lead and enhance the local invasion of cancer cells in lung adenocarcinoma. *Int. J. Cancer* 137 784–796. 10.1002/ijc.29464 25648219

[B90] Ngo M. A. Muller A. Li Y. Neumann S. Tian G. Dixon I. M. (2014). Human mesenchymal stem cells express a myofibroblastic phenotype in vitro: comparison to human cardiac myofibroblasts. *Mol. Cell Biochem.* 392 187–204. 10.1007/s11010-014-2030-6 24691634

[B91] Nguyen X. X. Muhammad L. Nietert P. J. Feghali-Bostwick C. (2018). IGFBP-5 promotes fibrosis via increasing its own expression and that of other pro-fibrotic mediators. *Front. Endocrinol.* 9:601. 10.3389/fendo.2018.00601 30374330 PMC6196226

[B92] Ni W. D. Yang Z. T. Cui C. A. Cui Y. Fang L. Y. Xuan Y. H. (2017). Tenascin-C is a potential cancer-associated fibroblasts marker and predicts poor prognosis in prostate cancer. *Biochem. Biophys. Res. Commun.* 486 607–612. 10.1016/j.bbrc.2017.03.021 28341124

[B93] Nilsson M. Adamo H. Bergh A. Halin Bergstrom S. (2016). Inhibition of lysyl oxidase and lysyl oxidase-like enzymes has tumour-promoting and tumour-suppressing roles in experimental prostate cancer. *Sci. Rep.* 6:19608. 10.1038/srep19608 26804196 PMC4726263

[B94] Nishio K. Motozawa K. Omagari D. Gojoubori T. Ikeda T. Asano M. (2016). Comparison of MMP2 and MMP9 expression levels between primary and metastatic regions of oral squamous cell carcinoma. *J. Oral Sci.* 58 59–65. 10.2334/josnusd.58.59 27021541

[B95] Oguic R. Mozetic V. Cini Tesar E. Fuckar Cupic D. Mustac E. Dordevic G. (2014). Matrix metalloproteinases 2 and 9 immunoexpression in prostate carcinoma at the positive margin of radical prostatectomy specimens. *Patholog. Res. Int.* 2014:262195. 10.1155/2014/262195 25097794 PMC4109076

[B96] Ohlund D. Handly-Santana A. Biffi G. Elyada E. Almeida A. S. Ponz-Sarvise M. (2017). Distinct populations of inflammatory fibroblasts and myofibroblasts in pancreatic cancer. *J. Exp. Med.* 214 579–596. 10.1084/jem.20162024 28232471 PMC5339682

[B97] Organ S. L. Tsao M. S. (2011). An overview of the c-MET signaling pathway. *Ther. Adv. Med. Oncol.* 3(Suppl. 1) S7–S19. 10.1177/1758834011422556 22128289 PMC3225017

[B98] Orimo A. Gupta P. B. Sgroi D. C. Arenzana-Seisdedos F. Delaunay T. Naeem R. (2005). Stromal fibroblasts present in invasive human breast carcinomas promote tumor growth and angiogenesis through elevated SDF-1/CXCL12 secretion. *Cell* 121 335–348. 10.1016/j.cell.2005.02.034 15882617

[B99] Ostermann E. Garin-Chesa P. Heider K. H. Kalat M. Lamche H. Puri C. (2008). Effective immunoconjugate therapy in cancer models targeting a serine protease of tumor fibroblasts. *Clin. Cancer Res.* 14 4584–4592. 10.1158/1078-0432.CCR-07-5211 18628473

[B100] Ozdemir B. C. Pentcheva-Hoang T. Carstens J. L. Zheng X. Wu C. C. Simpson T. R. (2014). Depletion of carcinoma-associated fibroblasts and fibrosis induces immunosuppression and accelerates pancreas cancer with reduced survival. *Cancer Cell* 25 719–734. 10.1016/j.ccr.2014.04.005 24856586 PMC4180632

[B101] Pankov R. Yamada K. M. (2002). Fibronectin at a glance. *J. Cell Sci.* 115 3861–3863. 10.1242/jcs.00059 12244123

[B102] Park J. S. Lee J. H. Lee Y. S. Kim J. K. Dong S. M. Yoon D. S. (2016). Emerging role of LOXL2 in the promotion of pancreas cancer metastasis. *Oncotarget* 7 42539–42552. 10.18632/oncotarget.9918 27285767 PMC5173154

[B103] Paszek M. J. Zahir N. Johnson K. R. Lakins J. N. Rozenberg G. I. Gefen A. (2005). Tensional homeostasis and the malignant phenotype. *Cancer Cell* 8 241–254. 10.1016/j.ccr.2005.08.010 16169468

[B104] Petersen O. W. Nielsen H. L. Gudjonsson T. Villadsen R. Ronnov-Jessen L. Bissell M. J. (2001). The plasticity of human breast carcinoma cells is more than epithelial to mesenchymal conversion. *Breast Cancer Res.* 3 213–217. 11434871 10.1186/bcr298 PMC138684

[B105] Philippeos C. Telerman S. B. Oules B. Pisco A. O. Shaw T. J. Elgueta R. (2018). Spatial and single-cell transcriptional profiling identifies functionally distinct human dermal fibroblast subpopulations. *J. Invest. Dermatol.* 138 811–825. 10.1016/j.jid.2018.01.016 29391249 PMC5869055

[B106] Pickup M. W. Laklai H. Acerbi I. Owens P. Gorska A. E. Chytil A. (2013). Stromally derived lysyl oxidase promotes metastasis of transforming growth factor-beta-deficient mouse mammary carcinomas. *Cancer Res.* 73 5336–5346. 10.1158/0008-5472.CAN-13-0012 23856251 PMC3766496

[B107] Pidsley R. Lawrence M. G. Zotenko E. Niranjan B. Statham A. Song J. (2018). Enduring epigenetic landmarks define the cancer microenvironment. *Genome Res.* 28 625–638. 10.1101/gr.229070.117 29650553 PMC5932604

[B108] Puram S. V. Tirosh I. Parikh A. S. Patel A. P. Yizhak K. Gillespie S. (2017). Single-cell transcriptomic analysis of primary and metastatic tumor ecosystems in head and neck cancer. *Cell* 171 1611–1624.e24. 10.1016/j.cell.2017.10.044 29198524 PMC5878932

[B109] Quante M. Tu S. P. Tomita H. Gonda T. Wang S. S. Takashi S. (2011). Bone marrow-derived myofibroblasts contribute to the mesenchymal stem cell niche and promote tumor growth. *Cancer Cell* 19 257–272. 10.1016/j.ccr.2011.01.020 21316604 PMC3060401

[B110] Ramos E. A. Silva C. T. Manica G. C. Pereira I. T. Klassen L. M. Ribeiro E. M. (2016). Worse prognosis in breast cancer patients can be predicted by immunohistochemical analysis of positive MMP-2 and negative estrogen and progesterone receptors. *Rev. Assoc. Med. Bras.* 62 774–781. 10.1590/1806-9282.62.08.774 27992019

[B111] Rao S. Rao J. Bm J. Vk V. (2016). Mysterious myofibroblast: a cell with diverse origin and multiple functions. *J. Interdiscip. Histopathol.* 5 12–17.

[B112] Robert F. Gordon M. S. Rosen L. S. Mendelson D. S. Mulay M. Adams B. J. (2010). Final results from a phase I study of TRC093 (humanized anti-cleaved collagen antibody) in patients with solid cancer. *J. Clin. Oncol.* 28 3038–3038. 10.1200/jco.2010.28.15_suppl.3038

[B113] Ronnov-Jessen L. Petersen O. W. (1993). Induction of alpha-smooth muscle actin by transforming growth factor-beta 1 in quiescent human breast gland fibroblasts. Implications for myofibroblast generation in breast neoplasia. *Lab. Invest.* 68 696–707. 8515656

[B114] Salmenpera P. Kankuri E. Bizik J. Siren V. Virtanen I. Takahashi S. (2008). Formation and activation of fibroblast spheroids depend on fibronectin-integrin interaction. *Exp. Cell Res.* 314 3444–3452. 10.1016/j.yexcr.2008.09.004 18824166

[B115] Schafer M. Werner S. (2008). Cancer as an overhealing wound: an old hypothesis revisited. *Nat. Rev. Mol. Cell Biol.* 9 628–638. 10.1038/nrm2455 18628784

[B116] Schutze F. Rohrig F. Vorlova S. Gatzner S. Kuhn A. Ergun S. (2015). Inhibition of lysyl oxidases improves drug diffusion and increases efficacy of cytotoxic treatment in 3D tumor models. *Sci. Rep.* 5:17576. 10.1038/srep17576 26620400 PMC4665164

[B117] Scott A. M. Wiseman G. Welt S. Adjei A. Lee F. T. Hopkins W. (2003). A Phase I dose-escalation study of sibrotuzumab in patients with advanced or metastatic fibroblast activation protein-positive cancer. *Clin. Cancer Res.* 9 1639–1647. 12738716

[B118] Serres E. Debarbieux F. Stanchi F. Maggiorella L. Grall D. Turchi L. (2014). Fibronectin expression in glioblastomas promotes cell cohesion, collective invasion of basement membrane in vitro and orthotopic tumor growth in mice. *Oncogene* 33 3451–3462. 10.1038/onc.2013.305 23912459

[B119] Shan S. Lv Q. Zhao Y. Liu C. Sun Y. Xi K. (2015). Wnt/beta-catenin pathway is required for epithelial to mesenchymal transition in CXCL12 over expressed breast cancer cells. *Int. J. Clin. Exp. Pathol.* 8 12357–12367. 26722422 PMC4680367

[B120] Shattil S. J. Kim C. Ginsberg M. H. (2010). The final steps of integrin activation: the end game. *Nat. Rev. Mol. Cell Biol.* 11 288–300. 10.1038/nrm2871 20308986 PMC3929966

[B121] Shiga K. Hara M. Nagasaki T. Sato T. Takahashi H. Takeyama H. (2015). Cancer-associated fibroblasts: their characteristics and their roles in tumor growth. *Cancers* 7 2443–2458. 10.3390/cancers7040902 26690480 PMC4695902

[B122] Spector I. Honig H. Kawada N. Nagler A. Genin O. Pines M. (2010). Inhibition of pancreatic stellate cell activation by halofuginone prevents pancreatic xenograft tumor development. *Pancreas* 39 1008–1015. 10.1097/MPA.0b013e3181da8aa3 20442678

[B123] Stanciu A. E. Zamfir-Chiru-Anton A. Stanciu M. M. Popescu C. R. Gheorghe D. C. (2016). Serum level of matrix metalloproteinase-9 in patients with head and neck squamous cell carcinoma. *Clin. Lab.* 62 1569–1574. 10.7754/Clin.Lab.2016.160139 28164611

[B124] Stenman S. Vaheri A. (1981). Fibronectin in human solid tumors. *Int. J. Cancer* 27 427–435. 7024140 10.1002/ijc.2910270403

[B125] Straussman R. Morikawa T. Shee K. Barzily-Rokni M. Qian Z. R. Du J. (2012). Tumour micro-environment elicits innate resistance to RAF inhibitors through HGF secretion. *Nature* 487 500–504. 10.1038/nature11183 22763439 PMC3711467

[B126] Sugihara H. Ishimoto T. Yasuda T. Izumi D. Eto K. Sawayama H. (2015). Cancer-associated fibroblast-derived CXCL12 causes tumor progression in adenocarcinoma of the esophagogastric junction. *Med. Oncol.* 32:618. 10.1007/s12032-015-0618-7 25920609

[B127] Sun Q. Zhao C. Xia L. He Z. Lu Z. Liu C. (2014). High expression of matrix metalloproteinase-9 indicates poor prognosis in human hilar cholangiocarcinoma. *Int. J. Clin. Exp. Pathol.* 7 6157–6164. 25337264 PMC4203235

[B128] Tabib T. Morse C. Wang T. Chen W. Lafyatis R. (2018). SFRP2/DPP4 and FMO1/LSP1 define major fibroblast populations in human skin. *J. Invest. Dermatol.* 138 802–810. 10.1016/j.jid.2017.09.045 29080679 PMC7444611

[B129] Tabouret E. Bertucci F. Pierga J. Y. Petit T. Levy C. Ferrero J. M. (2016). MMP2 and MMP9 serum levels are associated with favorable outcome in patients with inflammatory breast cancer treated with bevacizumab-based neoadjuvant chemotherapy in the BEVERLY-2 study. *Oncotarget* 7 18531–18540. 10.18632/oncotarget.7612 26921265 PMC4951307

[B130] Tabouret E. Boudouresque F. Barrie M. Matta M. Boucard C. Loundou A. (2014). Association of matrix metalloproteinase 2 plasma level with response and survival in patients treated with bevacizumab for recurrent high-grade glioma. *Neuro Oncol.* 16 392–399. 10.1093/neuonc/not226 24327581 PMC3922517

[B131] Taras D. Blanc J. F. Rullier A. Dugot-Senant N. Laurendeau I. Bieche I. (2006). Halofuginone suppresses the lung metastasis of chemically induced hepatocellular carcinoma in rats through MMP inhibition. *Neoplasia* 8 312–318. 10.1593/neo.05796 16756723 PMC1600678

[B132] Tarhini A. A. Rafique I. Floros T. Tran P. Gooding W. E. Villaruz L. C. (2017). Phase 1/2 study of rilotumumab (AMG 102), a hepatocyte growth factor inhibitor, and erlotinib in patients with advanced non-small cell lung cancer. *Cancer* 123 2936–2944. 10.1002/cncr.30717 28472537 PMC5517339

[B133] Teng F. Tian W. Y. Wang Y. M. Zhang Y. F. Guo F. Zhao J. (2016). Cancer-associated fibroblasts promote the progression of endometrial cancer via the SDF-1/CXCR4 axis. *J. Hematol. Oncol.* 9:8. 10.1186/s13045-015-0231-4 26851944 PMC4744391

[B134] Torres S. Garcia-Palmero I. Herrera M. Bartolome R. A. Pena C. Fernandez-Acenero M. J. (2015). LOXL2 is highly expressed in cancer-associated fibroblasts and associates to poor colon cancer survival. *Clin. Cancer Res.* 21 4892–4902. 10.1158/1078-0432.CCR-14-3096 26206869

[B135] Tran H. C. Wan Z. Sheard M. A. Sun J. Jackson J. R. Malvar J. (2017). TGFbetaR1 blockade with galunisertib (LY2157299) enhances anti-neuroblastoma activity of the anti-GD2 antibody dinutuximab (ch14.18) with natural killer cells. *Clin. Cancer Res.* 23 804–813. 10.1158/1078-0432.CCR-16-1743 27756784 PMC5361893

[B136] Tucker R. P. Chiquet-Ehrismann R. (2015). Tenascin-C: its functions as an integrin ligand. *Int. J. Biochem. Cell Biol.* 65 165–168. 10.1016/j.biocel.2015.06.003 26055518

[B137] Tyan S. W. Kuo W. H. Huang C. K. Pan C. C. Shew J. Y. Chang K. J. (2011). Breast cancer cells induce cancer-associated fibroblasts to secrete hepatocyte growth factor to enhance breast tumorigenesis. *PLoS One* 6:e15313. 10.1371/journal.pone.0015313 21249190 PMC3020942

[B138] Valkenburg K. C. de Groot A. E. Pienta K. J. (2018). Targeting the tumour stroma to improve cancer therapy. *Nat. Rev. Clin. Oncol.* 15 366–381. 10.1038/s41571-018-0007-1 29651130 PMC5960434

[B139] van Helvert S. Storm C. Friedl P. (2018). Mechanoreciprocity in cell migration. *Nat. Cell Biol.* 20 8–20. 10.1038/s41556-017-0012-0 29269951 PMC5943039

[B140] Vandenbroucke R. E. Libert C. (2014). Is there new hope for therapeutic matrix metalloproteinase inhibition? *Nat. Rev. Drug Discov.* 13 904–927. 10.1038/nrd4390 25376097

[B141] Wang C. Ma H. X. Jin M. S. Zou Y. B. Teng Y. L. Tian Z. (2014). Association of matrix metalloproteinase (MMP)-2 and -9 expression with extra-gastrointestinal stromal tumor metastasis. *Asian Pac. J. Cancer Prev.* 15 4187–4192. 24935368 10.7314/apjcp.2014.15.10.4187

[B142] Wang J. Chen H. Seth A. McCulloch C. A. (2003). Mechanical force regulation of myofibroblast differentiation in cardiac fibroblasts. *Am. J. Physiol. Heart Circ. Physiol.* 285 H1871–H1881. 10.1152/ajpheart.00387.2003 12842814

[B143] Wang M. Wang T. Liu S. Yoshida D. Teramoto A. (2003). The expression of matrix metalloproteinase-2 and -9 in human gliomas of different pathological grades. *Brain Tumor Pathol.* 20 65–72. 14756443 10.1007/BF02483449

[B144] Wang T. H. Hsia S. M. Shieh T. M. (2016). Lysyl oxidase and the tumor microenvironment. *Int J Mol Sci* 18:62. 10.3390/ijms18010062 28036074 PMC5297697

[B145] Wells R. G. Schwabe R. F. (2015). Origin and function of myofibroblasts in the liver. *Semin. Liver Dis.* 35 97–106. 10.1055/s-0035-1550061 25974896

[B146] Wen X. Liu Y. Bai Y. Li M. Fu Q. Zheng Y. (2018). LOXL2, a copper-dependent monoamine oxidase, activates lung fibroblasts through the TGF-beta/Smad pathway. *Int. J. Mol. Med.* 42 3530–3541. 10.3892/ijmm.2018.3927 30320382

[B147] Wu Q. W. Yang Q. M. Huang Y. F. She H. Q. Liang J. Yang Q. L. (2014). Expression and clinical significance of matrix metalloproteinase-9 in lymphatic invasiveness and metastasis of breast cancer. *PLoS One* 9:e97804. 10.1371/journal.pone.0097804 24845596 PMC4028268

[B148] Wu X. Chen X. Zhou Q. Li P. Yu B. Li J. (2013). Hepatocyte growth factor activates tumor stromal fibroblasts to promote tumorigenesis in gastric cancer. *Cancer Lett.* 335 128–135. 10.1016/j.canlet.2013.02.002 23402812

[B149] Xing F. Saidou J. Watabe K. (2010). Cancer associated fibroblasts (CAFs) in tumor microenvironment. *Front. Biosci.* 15 166–179. 10.2741/3613 PMC2905156 20036813

[B150] Yamada M. Sekiguchi K. (2015). “Chapter six - molecular basis of laminin–integrin interactions,” in *Current Topics in Membranes* ed. Miner J. H. (Cambridge, MA: Academic Press) 197–229. 10.1016/bs.ctm.2015.07.002 26610915

[B151] Yang X. Lin Y. Shi Y. Li B. Liu W. Yin W. (2016). FAP promotes immunosuppression by cancer-associated fibroblasts in the tumor microenvironment via STAT3-CCL2 signaling. *Cancer Res.* 76 4124–4135. 10.1158/0008-5472.CAN-15-2973 27216177

[B152] Yari K. Rahimi Z. Moradi M. T. Rahimi Z. (2014). The MMP-2 -735 C allele is a risk factor for susceptibility to breast cancer. *Asian Pac. J. Cancer Prev.* 15 6199–6203. 25124598 10.7314/apjcp.2014.15.15.6199

[B153] Yeung T. L. Leung C. S. Wong K. K. Samimi G. Thompson M. S. Liu J. (2013). TGF-beta modulates ovarian cancer invasion by upregulating CAF-derived versican in the tumor microenvironment. *Cancer Res.* 73 5016–5028. 10.1158/0008-5472.CAN-13-0023 23824740 PMC3745588

[B154] Yoshimura H. Michishita M. Ohkusu-Tsukada K. Matsuda Y. Ishiwata T. Naito Z. (2015). Cellular sources of tenascin-C in canine mammary carcinomas. *Vet. Pathol.* 52 92–96. 10.1177/0300985814522817 24565830

[B155] Yousef E. M. Tahir M. R. St-Pierre Y. Gaboury L. A. (2014). MMP-9 expression varies according to molecular subtypes of breast cancer. *BMC Cancer* 14:609. 10.1186/1471-2407-14-609 25151367 PMC4150970

[B156] Yu Y. Ding Z. Jian H. Shen L. Zhu L. Lu S. (2016). Prognostic value of MMP9 activity level in resected stage I B lung adenocarcinoma. *Cancer Med.* 5 2323–2331. 10.1002/cam4.821 27456862 PMC5055171

[B157] Yu Y. Xiao C. H. Tan L. D. Wang Q. S. Li X. Q. Feng Y. M. (2014). Cancer-associated fibroblasts induce epithelial-mesenchymal transition of breast cancer cells through paracrine TGF-beta signalling. *Br. J. Cancer* 110 724–732. 10.1038/bjc.2013.768 24335925 PMC3915130

[B158] Zeisberg E. M. Potenta S. Xie L. Zeisberg M. Kalluri R. (2007a). Discovery of endothelial to mesenchymal transition as a source for carcinoma-associated fibroblasts. *Cancer Res.* 67 10123–10128. 10.1158/0008-5472.CAN-07-3127 17974953

[B159] Zeisberg E. M. Tarnavski O. Zeisberg M. Dorfman A. L. McMullen J. R. Gustafsson E. (2007b). Endothelial-to-mesenchymal transition contributes to cardiac fibrosis. *Nat. Med.* 13 952–961. 10.1038/nm1613 17660828

[B160] Zhang M. Zhang X. (2015). Association of MMP-2 expression and prognosis in osteosarcoma patients. *Int. J. Clin. Exp. Pathol.* 8 14965–14970. 26823829 PMC4713615

[B161] Zhang X. Schonrogge M. Eichberg J. Wendt E. H. U. Kumstel S. Stenzel J. (2018). Blocking autophagy in cancer-associated fibroblasts supports chemotherapy of pancreatic cancer cells. *Front. Oncol.* 8:590. 10.3389/fonc.2018.00590 30568920 PMC6290725

[B162] Zhang Y. Shen Y. Cao B. Yan A. Ji H. (2015). Elevated expression levels of androgen receptors and matrix metalloproteinase-2 and -9 in 30 cases of hepatocellular carcinoma compared with adjacent tissues as predictors of cancer invasion and staging. *Exp. Ther. Med.* 9 905–908. 10.3892/etm.2014.2150 25667651 PMC4316905

[B163] Zhu C. Q. Popova S. N. Brown E. R. Barsyte-Lovejoy D. Navab R. Shih W. (2007). Integrin alpha 11 regulates IGF2 expression in fibroblasts to enhance tumorigenicity of human non-small-cell lung cancer cells. *Proc. Natl. Acad. Sci. U.S.A.* 104 11754–11759. 10.1073/pnas.0703040104 17600088 PMC1913903

